# Maternal, fetal and perinatal factors associated with necrotizing enterocolitis in Sweden. A national case-control study

**DOI:** 10.1371/journal.pone.0194352

**Published:** 2018-03-23

**Authors:** Margareta Ahle, Peder Drott, Anders Elfvin, Roland E. Andersson

**Affiliations:** 1 Department of Radiology and Department of Medical and Health Sciences, Linköping University, Linköping, Sweden; 2 Division of Surgery, Department of Clinical and Experimental Medicine, Linköping University, Linköping, Sweden; 3 Department of Pediatrics, Institution of Clinical Sciences, Sahlgrenska Academy, University of Gothenburg, Gothenburg, Sweden; 4 Department of Surgery, Ryhov County Hospital, Jönköping, Sweden; Hopital Robert Debre, FRANCE

## Abstract

**Objective:**

To analyze associations of maternal, fetal, gestational, and perinatal factors with necrotizing enterocolitis in a matched case-control study based on routinely collected, nationwide register data.

**Study design:**

All infants born in 1987 through 2009 with a diagnosis of necrotizing enterocolitis in any of the Swedish national health care registers were identified. For each case up to 6 controls, matched for birth year and gestational age, were selected. The resulting study population consisted of 720 cases and 3,567 controls. Information on socioeconomic data about the mother, maternal morbidity, pregnancy related diagnoses, perinatal diagnoses of the infant, and procedures in the perinatal period, was obtained for all cases and controls and analyzed with univariable and multivariable logistic regressions for the whole study population as well as for subgroups according to gestational age.

**Results:**

In the study population as a whole, we found independent positive associations with necrotizing enterocolitis for isoimmunization, fetal distress, cesarean section, neonatal bacterial infection including sepsis, erythrocyte transfusion, persistent ductus arteriosus, cardiac malformation, gastrointestinal malformation, and chromosomal abnormality. Negative associations were found for maternal weight, preeclampsia, maternal urinary infection, premature rupture of the membranes, and birthweight. Different patterns of associations were seen in the subgroups of different gestational age.

**Conclusion:**

With some interesting exceptions, especially in negative associations, the results of this large, population based study, are in keeping with earlier studies. Although restrained by the limitations of register data, the findings mirror conceivable pathophysiological processes and underline that NEC is a multifactorial disease.

## Introduction

Necrotizing enterocolitis (NEC) remains a challenge in neonatal care. The incidence rate of NEC in Sweden during 1987 through 2009 was 0.34 in 1,000 live births, with an increasing trend, partly explained by increased survival of the most premature but also seen in higher gestational ages (GA)[1].

The etiology is multifactorial and may differ according to the degree of maturity of the patient[2]. Prematurity and low birthweight are the most consistent predisposing factors, whereas other risk factors vary with GA,[3,4] as well as between study populations, and the results of previous studies are sometimes contradictory.

Pathophysiologically, the complex interactions of the innate immunity system and colonizing bacteria, pro-inflammatory factors and modulatory systems, are deranged[5,6]. The immaturity of intestinal motility and digestion, structural and biochemical barrier functions as well as circulatory regulation contribute to the vulnerability[5,6]. The result is hemorrhagic and necrotizing inflammation, bacterial overgrowth and translocation of bacteria to the intestinal wall and systemic circulation[7].

The presence of bacteria is thus an important prerequisite in the pathogenesis of NEC, but their role as contagions is disputable. Reports of seasonal variation in incident rates, episodic outbreaks and clustering of NEC,[1,8] however, suggest a role for transmissible infectious agents or other environmental factors.

### Objective of the study

The aim of the present case control study is to analyze differences in maternal, fetal, gestational, and perinatal factors among NEC cases and matched controls from routinely collected register data from Sweden 1987 through 2009, in order to identify associations of these factors with NEC.

## Material and methods

Data were obtained from the National Patient Register (NPR), the Swedish Medical Birth Register (MBR), and the National Cause of Death Register (NCD). All infants born 1987 through 2009 with a registered diagnosis of NEC, according to the World Health Organization (WHO) International Classification of Disease system, ninth or tenth revision (ICD9 or 10), were identified.

A total of 2,399 episodes, i.e. admissions in the NPR and registrations in the MBR and NCD, with a diagnosis of NEC were found. 676 episodes were excluded because of missing personal identification number[9]. 1,723 episodes remained, belonging to 794 individuals with complete identity information, allowing linkage between the registers. Note that several episodes may belong to one individual. 74 of these cases could not be found in the MBR, leaving 720 cases. For each case, we aimed to identify 6 randomly selected controls, matched for birth year and GA. Due to limited number of available controls in some strata, we failed to reach the desired number of controls for some cases, especially in GA < 32 w. The goal of six controls per case was met in 70% of the cases. The mean number of controls per case was 3.9 in infants with GA<28 w and 6.0 for the term infants. The resulting study population consisted of 720 cases and 3,656 controls.

Information on morbidity and pregnancy related diagnoses of the mother, perinatal diagnoses of the child, as well as codes of procedures in the perinatal period, was obtained from the MBR, NPR and NCD for all cases and controls. Socioeconomic data about the mothers, such as country of birth, family type (living with the father of the child or not), length of education, work status, and income was obtained from Statistics Sweden. Hospitalizations were considered relevant in infants if the admission occurred within four weeks of birth.

As predisposing factors are known to vary with the degree of prematurity, all analyzes were made in the entire study population and in subgroups of GA defined according to the definitions of the World Health Organization (WHO) International Classification of Disease system, tenth revision (ICD 10). Because of the low frequency of NEC among infants with a GA >42 weeks, they were merged with the full-term group.

Descriptive statistics are provided for cases and controls. To identify factors associated with NEC, univariable and multivariable logistic regression was used, reporting results as odd’s ratio (OR) with a 95% confidence interval (CI). The matching variables, i.e. GA and year of birth were included in all regressions. Some children that died early may have died before they developed NEC. This competing risk was treated by including a variable for 7 days survival as a covariate in all regressions.

We used Least Angle Regression (LARS) to identify potential variables to include in the final multivariable models. We made separate LARS analyses in each subgroup of GA and kept all variables that were selected for any of these groups. Variables deemed to be spuriously associated with NEC on pathophysiological grounds, variables showing signs of multicollinearity, and variables giving unstable models because of few cases in some strata, were sought for and removed. We finally added variables that have previously been reported as risk factors. We included all these variables in all analyses to avoid under specification bias.

Some missing values were found in birthweight (n = 90), length at birth (n = 932), Apgar score at 1 minute (n = 174), Apgar score at 5 minutes (n = 197), Apgar score at 10 minutes (n = 627), maternal weight (n = 1,245), and maternal disposable income (n = 47). Missing values were supplemented through multivariate imputation using chained equations, using predictive mean matching for continuous variables, and ordered logistic regression for ordinal variables[10]. We made sensitivity analyses to analyze the impact of imputed values that were not missing at random, the inclusion of a covariate for >7 days survival, and the effect of the long study period. We used Stata 15 for all statistical analyses (StataCorp. 2017. Stata Statistical Software: Release 15. College Station, TX: StataCorp LLC.

No consent was obtained from the participants, since all data were analyzed anonymously. The study was approved by the Regional Ethical Review Board of Linköping.

## Results

The characteristics of the cases and controls are presented in Table 1. The difference in GA is explained by the unequal number of controls in the stratas, with fewer controls in the most premature infants. The difference in 7 days survival, a prerequisite for developing NEC, is evident in GA < 28 w. All the following results are adjusted for these two factors and year of birth. The uni- and multivariable analyses of the associations with NEC for the entire study population, are presented in Table 2, the univariable analyses in subgroups according to GA in S1 Table, and the multivariable analyses in subsets in Table 3.

**Table 1:** Basic characteristics of cases and controls.

Basic characteristics			Cases N = 720		Controls. N = 3656	
Numbers						
	Full term	n	138		828	
	GA 32–36 w	n	117		701	
	GA 28–31 w	n	196		1065	
	GA < 28 w	n	269		1062	
Mortality			at 7 days	at 28 days	at 7 days	at 28 days
	Overall	n (%)	39 (5%)	107 (15%)	258 (7%)	305 (8%)
	Full term	n (%)	2 (1%)	5 (4%)	0	0
	GA 32–36 w	n (%)	6 (5%)	8 (7%)	13 (2%)	17 (2%)
	GA 28–31 w	n (%)	13 (7%)	33 (17%)	53 (5%)	60 (6%)
	GA < 28 w	n (%)	18(7%)	61 (23%)	192 (18%)	228 (22%)
Maternal age (years)		mean	29.9		29.5	
Maternal weight (kg)		mean	65.6		67.1	
Maternal smoking		n (%)	106 (15%)		638 (17%)	
Maternal education > 12 years		n (%)	251 (35%)		1137 (31%)	
Maternal unemployment		n (%)	95 (13%)		587 (16%)	
Maternal disposable income (100 E)		mean	143		131	
Born in Stockholm		n (%)	255 (35%)		804 (22%)	
First born		n (%)	363 (50%)		1858 (51%)	
Male sex		n (%)	394 (55%)		2006 (55%)	
Gestational age (days)		mean	216.5		222.5	
Birth weight (g)		mean	1618		1887	
	Full term		3337		3476	
	GA 32–36 w		2148		2328	
	GA 28–31 w		1260		1355	
	GA < 28 w		800		860	
Length (cm)		mean	40.1		42.4	
Ponderal index		mean	23.6		24.2	
Head circumference (cm)		mean	28.3		29.9	
Small for gestational age		n (%)	176 (24%)		596 (16%)	
Spontaneously starting delivery		n (%)	299 (42%)		1767 (48%)	
Cesarean section		n (%)	409 (57%)		1560 (43%)	
Apgar at 1 minute		mean	6.3		6.9	
Apgar at 5 minutes		mean	8.0		8.4	
Apgar at 10 minutes		mean	8.8		9.0	

CI − Confidence Interval; OR–Odds Ratio; GA–Gestational age

**Table 2:** Associations of maternal, gestational, fetal, and perinatal factors with NEC in all gestational ages, univariable and multivariable regressions.

	Cases, N = 720	Controls, N = 3656	Univariable regression				Multivariable regression			
	n (%)	n (%)	OR	95% CI		P	OR	95% CI		p
Maternal age (years)			**1.01**	1.00	1.03	**0.126**	**0.99**	0.97	1.01	**0.367**
Maternal weight (kg)			**0.99**	0.98	1.00	**0.004**	**0.99**	0.98	1.00	**0.035**
Maternal smoking	106 (15%)	638 (17%)	**0.84**	0.66	1.05	**0.123**	**0.82**	0.64	1.05	**0.122**
Education>12 y	251 (35%)	1137 (31%)	**1.17**	0.99	1.39	**0.066**	**1.03**	0.84	1.25	**0.787**
Maternal unemployment	95 (13%)	587 (16%)	**0.78**	0.62	0.99	**0.038**	**0.89**	0.69	1.14	**0.355**
Maternal disposable income (100 E)			**1.13**	1.03	1.24	**0.008**	**1.08**	0.98	1.20	**0.132**
Born in Stockholm	255 (35%)	804 (22%)	**1.95**	1.64	2.32	**<0.001**	**1.89**	1.56	2.29	**<0.001**
First born	363 (50%)	1858 (51%)	**0.96**	0.81	1.12	**0.579**	**0.87**	0.72	1.05	**0.144**
Maternal diabetes	19 (3%)	105 (3%)	**0.88**	0.53	1.44	**0.608**	**0.97**	0.57	1.64	**0.896**
Preeclampsia	144 (20%)	686 (19%)	**1.00**	0.82	1.23	**0.993**	**0.69**	0.53	0.89	**0.005**
Maternal urinary infection	68 (9%)	440 (12%)	**0.72**	0.55	0.95	**0.018**	**0.70**	0.53	0.94	**0.017**
Isoimmunization	40 (6%)	131 (4%)	**1.53**	1.06	2.21	**0.023**	**1.63**	1.10	2.43	**0.016**
Placental complications	139 (19%)	584 (16%)	**1.21**	0.98	1.49	**0.076**	**1.02**	0.81	1.29	**0.857**
Chorioamnionitis	185 (26%)	563 (15%)	**0.75**	0.52	1.09	**0.131**	**0.96**	0.64	1.45	**0.852**
PROM	36 (5%)	206 (6%)	**0.54**	0.44	0.68	**<0.001**	**0.63**	0.49	0.80	**<0.001**
Fetal distress	109 (15%)	831 (23%)	**1.86**	1.54	2.25	**<0.001**	**1.44**	1.15	1.80	**0.001**
Spontaneously starting delivery	299 (42%)	1767 (48%)	**0.73**	0.61	0.86	**<0.001**				
Cesarean section	409 (57%)	1560 (43%)	**1.65**	1.40	1.96	**<0.001**	**1.54**	1.25	1.89	**<0.001**
Apgar < 7 at 1 minute	180 (25%)	695 (19%)	**1.28**	1.05	1.55	**0.014**				
Apgar<7 at1 and 5 minutes	88 (12%)	331 (9%)	**1.26**	0.97	1.63	**0.082**	**1.11**	0.84	1.48	**0.467**
Apgar<7 at 1. 5. and 10 minutes	48 (7%)	231 (6%)	**1.08**	0.76	1.52	**0.682**				
Male sex	394 (55%)	2006 (55%)	**0.99**	0.84	1.16	**0.891**	**1.01**	0.85	1.20	**0.927**
Birth weight (100 g)			**0.93**	0.91	0.95	**<0.001**	**0.94**	0.90	0.97	**<0.001**
Length (cm)			**0.87**	0.84	0.90	**<0.001**				
Ponderal index			**1.00**	0.97	1.03	**0.973**	**1.02**	0.98	1.06	**0.333**
Head circumference			**0.89**	0.84	0.94	**<0.001**				
Small for gestational age	176 (24%)	596 (16%)	**1.56**	1.28	1.89	**<0.001**	**1.03**	0.77	1.34	**0.850**
Neonatal anemia	159 (22%)	680 (19%)	**1.03**	0.83	1.27	**0.803**	**0.93**	0.73	1.19	**0.533**
Neonatal icterus	305 (42%)	1752 (48%)	**0.59**	0.49	0.70	**<0.001**	**0.57**	0.47	0.70	**<0.001**
Bacterial infection including sepsis	290 (40%)	730 (20%)	**2.73**	2.25	3.31	**<0.001**	**2.54**	2.06	3.12	**<0.001**
Erythrocyte transfusion	158 (22%)	518 (14%)	**1.51**	1.22	1.89	**<0.001**	**1.34**	1.05	1.73	**0.021**
Persistent Ductus Arteriosus	204 (28%)	554 (15%)	**2.09**	1.68	2.58	**<0.001**	**1.70**	1.34	2.17	**<0.001**
Cardiac malformation	48 (7%)	123 (3%)	**1.97**	1.39	2.78	**<0.001**	**1.64**	1.12	2.41	**0.012**
Gastrointestinal malformation	22 (3%)	35 (1%)	**3.24**	1.88	5.57	**<0.001**	**2.74**	1.52	5.00	**0.001**
Chromosomal abnormality	19 (3%)	28 (1%)	**3.96**	2.18	7.19	**<0.001**	**2.55**	1.30	5.01	**0.006**
IRDS	311 (43%)	1185 (32%)	**1.45**	1.18	1.77	**<0.001**	**1.08**	0.86	1.35	**0.508**
Retinopathy of the premature	58 (8%)	166 (5%)	**1.51**	1.09	2.09	**0.013**	**1.21**	0.84	1.75	**0.297**
Intraventricular Hemorrhage	113 (16%)	305 (8%)	**1.87**	1.46	2.39	**<0.001**	**1.74**	1.32	2.28	**<0.001**
Bronchopulmonary Dysplasia	88 (12%)	372 (10%)	**0.97**	0.74	1.26	**0.804**	**0.61**	0.45	0.83	**0.002**

CI–Confidence Interval; IRDS–Infant Respiratory Distress Syndrome; OR–Odds Ratio; PROM–Premature Rupture of the Membranes

**Table 3:** Associations of maternal, gestational, fetal, and perinatal factors with NEC in subgroups according to gestational age, multivariable regression.

Multivariable regression, subgroups	Term				GA 32–36 w				GA 28–31 w				GA <28 w			
	OR	95% CI		p	OR	95% CI		p	OR	95% CI		p	OR	95% CI		P
Maternal age (years)	1.01	0.96	1.05	0.907	1.04	1.00	1.09	0.044	1.02	0.99	1.05	0.154	0.98	0.95	1.00	0.065
Maternal weight (kg)	1.00	0.98	1.02	0.985	1.00	0.98	1.03	0.861	0.99	0.97	1.00	0.063	0.99	0.97	1.00	0.040
Born in Stockholm	2.35	1.44	3.83	0.001	3.09	1.84	5.17	<0.001	1.90	1.31	2.77	0.001	1.69	1.22	2.33	0.002
Preeclampsia	0.77	0.30	1.95	0.579	0.63	0.33	1.22	0.172	0.66	0.43	1.02	0.059	0.93	0.58	1.50	0.766
Maternal diabetes	2.87	0.84	9.86	0.094	0.94	0.19	4.49	0.918	0.90	0.35	2.27	0.817	0.48	0.16	1.43	0.186
Maternal urinary infection	1.03	0.45	2.35	0.942	1.02	0.47	2.21	0.952	0.61	0.34	1.10	0.103	0.55	0.34	0.87	0.011
Fetal distress	3.54	1.88	6.67	<0.001	2.18	1.23	3.85	0.007	0.66	0.43	1.00	0.049	1.30	0.86	1.96	0.210
Chorioamnionitis													1.00	0.62	1.60	0.995
PROM					0.43	0.22	0.83	0.012	0.66	0.43	1.02	0.064	0.61	0.43	0.88	0.008
Isoimmunization	3.96	1.40	11.23	0.010	0.71	0.24	2.07	0.529	2.50	1.24	5.03	0.011	1.09	0.52	2.22	0.838
Cesarean section	3.72	2.15	6.45	<0.001	2.07	1.23	3.48	0.006	1.42	0.96	2.09	0.077	0.93	0.66	1.29	0.649
Neonatal anemia					1.80	0.68	4.76	0.235	0.99	0.65	1.51	0.979	0.64	0.46	0.90	0.011
Apgar<7 at 1 and 5 minutes	4.07	0.73	22.53	0.108	3.20	1.26	8.08	0.014	1.17	0.66	2.06	0.593	0.90	0.62	1.29	0.554
Neonatal icterus	1.77	0.68	4.58	0.24	0.46	0.27	0.78	0.004	0.45	0.32	0.65	<0.001	0.75	0.54	1.03	0.071
Bacterial infection including sepsis	30.77	11.69	81.01	<0.001	9.00	4.64	17.44	<0.001	2.67	1.87	3.80	<0.001	1.31	0.97	1.77	0.080
Erythrocyte transfusion					5.21	2.03	13.34	0.001	1.89	1.22	2.93	0.005	0.94	0.67	1.33	0.732
Birth weight (100 g)	0.99	0.92	1.06	0.692	0.95	0.87	1.04	0.254	0.83	0.75	0.91	<0.001	0.83	0.73	0.96	0.009
Ponderal index	1.04	0.92	1.17	0.524	1.00	0.90	1.10	0.973	1.03	0.95	1.11	0.521	1.04	0.98	1.11	0.081
Small for gestational age	6.34	2.26	17.79	0.001	1.25	0.54	2.89	0.607	0.63	0.36	1.12	0.117	0.70	0.39	1.24	0.221
Male sex	0.99	0.61	1.59	0.957	1.05	0.65	1.69	0.840	1.07	0.76	1.50	0.696	0.97	0.73	1.30	0.863
Gastrointestinal malformation					1.74	0.35	8.55	0.498	1.03	0.33	3.25	0.953	3.46	1.16	10.31	0.026
PDA	21.32	3.40	133.69	0.001	3.22	1.20	8.66	0.020	1.75	1.12	2.71	0.013	1.27	0.93	1.73	0.137
Cardiac malformation	8.30	2.61	26.40	<0.001	4.48	1.78	11.25	0.001	0.80	0.34	1.90	0.616	0.61	0.28	1.30	0.196

CI–Confidence Interval; OR–Odds Ratio; PDA–Persistent Ductus Arteriosus; PROM–Premature Rupture of the Membranes; GA–Gestational age

In GA < 28 w, only lower birth weight, despite matched GA, and gastrointestinal malformation were positively associated with NEC. Negative associations with NEC were noted in this group for maternal urinary infection during pregnancy, premature rupture of the membranes (PROM), and neonatal anemia. In GA 28–31 w, low birth weight had a similar, effect as in < 28 w, but in addition this group followed the more mature groups in positive associations of NEC with isoimmunization, fetal distress, persistent ductus arteriosus (PDA), bacterial infection/sepsis, and erythrocyte transfusion. As for comorbidities typical of prematurity, infant respiratory distress syndrome (IRDS), retinopathy of the premature (ROP), intracranial bleeding, predominantly intraventricular bleeding (IVH), and chronic lung disease/bronchopulmonary dysplasia (BPD) were all more common among NEC cases than controls with a positive relationship in the univariable analysis for all except BPD. In the multivariable model only the positive association of NEC with IVH remained statistically significant, whereas a negative association with BPD emerged.

In GA > 31 w, positive associations with a diagnosis of cardiac malformations, cesarean section (CS), chromosomal abnormalities, and Apgar < 7 were added. More pronounced distress, i.e. Apgar < 4, did not further influence the risk. A positive association with being small for gestational age (SGA) was seen in term infants only.

There were independent, negative associations of NEC with maternal weight and a maternal diagnosis of pre-eclampsia (PE), significant only in the total study population, but with essentially uniform tendencies in the subgroups. Chromosomal abnormalities were positively associated with NEC in the population as a whole but because of low numbers not included in the subgroup models.

Analysis of the birth countries of the mothers did not show any relationship to NEC. Among socioeconomic factors, the only factor that remained significant in the multivariable model was being born in Stockholm. There were no significant associations with parity, smoking, maternal diabetes, chorioamnionitis or placental complications. Pregnancy related hypertensive disorders, apart from PE, were too few to be included in the models.

A discharge diagnosis of intestinal perforation in the newborn was found in 78 individuals, 64 cases and 14 controls, with a significant increasing trend over time as analyzed with poisson regression: Incidence rate ratio (IRR) 1,11 (95% CI 1,06–1,15) per year in the entire study population, p<0,001; IRR 1,10 (1,05–1,15) per year in cases, p<0,001, and 1,09 (0,996–1,9), p = 0,06, in controls.

The sensitivity analysis showed only small and nonsignificant differences at the third digit level when imputed variables were included in univariable analyses compared with complete case analyses. Separate multivariable analyses for the study period before and from year 2000 showed a significantly larger association of NEC with gastrointestinal malformations with a very large CI due to the few number of cases in each period. Excluding the covariat for >7 days survival resulted in nonsignificant changes at the second digit level in all variables except persistent ductus arteriosus which became significantly associated with NEC in both uni- and multivariate analyses in GA < 28 w, with an OR 1.49 (p = 0.010) in the multivariable analysis compared with the reported OR 1.27 (p = 0.137).

## Discussion

We found a complex pattern of associations between NEC and numerous factors related to the mother, pregnancy, infant and perinatal events. Similar to many previous studies, associations with NEC were more obvious in GA > 31 weeks, than in the more premature, which reflects the overriding effect of prematurity but also supports the notion that different mechanisms are involved at different levels of maturity.

### Maternal factors

We have no explanation for the slightly negative association of NEC with increasing maternal weight. Maternal obesity, as well as undernutrition, has been shown to increase the risk of several complications,[11,12] most of which are controlled for in the multivariable analysis. Maternal obesity, body composition and/or diet have also been shown to influence neonatal body composition [13] as well as maternal milk content of several macronutrients, bioactive substances,[14–16] and microbiota,[17] all of which could possibly influence the complex pathogenetic mechanisms of NEC.

In the multivariable model, no association of NEC with smoking, educational level, income, or unemployment was seen. The association with being born in Stockholm, the capital and largest city of Sweden, was noted in our previous study on incidence of NEC in Sweden[1] and interpreted as a higher tendency to assign the diagnosis.

### Pregnancy related factors

Fetal distress mirrors an antenatal asphyxic event, not surprisingly positively associated with NEC in GA > 31 w[3]. Isoimmunization is likely to predispose to a general vulnerability and can be speculated to contribute to imbalance of the developing immune system, which might precipitate NEC. Case reports and retrospective studies have suggested that treatment of hemolytic anemia with intravenous immunoglobulines may precipitate NEC in term and near term infants[18–20]. Whether the association of NEC with isoimmunization is linked to the condition itself or to its treatment, cannot be discerned from our data.

The negative association with maternal urinary infection and PROM, is somewhat surprising, especially as PROM, leading to chorioamnionitis, has been suggested as a risk factor of NEC[21,22]. A meta-analysis from 2013, however, found a tendency towards lower risk for NEC with a shorter course of antibiotics in mothers treated for PROM[23]. We speculate that low grade antenatal exposure to pathogens in maternal urinary infection or PROM, possibly balanced by a short course of antibiotics, may speed up the transfer of immunoglobulines to the fetus and maternal milk, which may in turn contribute to modulation of intestinal inflammatory responses in the neonate.

Another notable finding is the negative association of NEC with PE, which emerges in the multivariable analysis, significant only in the total study population, but with a uniform tendency in all sub-groups. The literature is inconclusive. A protective effect of maternal hypertensive disorders has been reported[4] but also positive associations of PE with NEC at least in some subgroups[24,25]. The number of NEC cases in our population was, however, considerably larger than in those studies, and many possible mediating factors were controlled for in the multivariable analysis. There is no obvious explanation for our finding, but negative associations of PE have also been reported with IVH[26] and ROP,[27] maybe above all mirroring the complexity of interactions in pregnancy and prematurity.

### Perinatal factors

Delivery by CS was associated with a greater incidence of NEC except in GA > 31 w. It made no difference if CS was elective or finalized an initially spontaneous delivery. Earlier results have been equivocal, reporting higher risk after CS[28], lower risk [29] or no difference[30]. CS is associated with lower diversity of the gut microbiota of the newborn and differences in dominant species compared to infants born by the vaginal route,[31] which may predispose for NEC[32,33]. In vaginal delivery, colonization of the infant gut is expected to result from the direct exposure to maternal vaginal and intestinal flora, but the composition of the microbiome in breast milk may also be altered after elective CS compared to normal labor[17].

Apgar scores less than 7 at five minutes were associated with increased NEC incidence in GA 32–36 w. Otherwise there was no association of low Apgar with NEC.

### Factors related to the infant

The negative association of birthweight with NEC, even in GA < 28 w, indicates that lower birthweight adds to the risk for NEC among infants of equal degree of prematurity.

Being SGA, according to the routine assessment at birth, was associated with NEC only for full term infants. Earlier results on the impact of being SGA on the risk of NEC in the premature is contradictory with no increased risk for NEC reported for very low birth weight infants in Taiwan,[34] in contrast to increased risk in preterm babies in the USA and Canada[35,36]. There was no association to ponderal index, which means that any growth restriction influencing the risk for NEC would be symmetrical,[37] which in turn is thought to reflect an early cause of growth restriction[38].

Male sex has been suggested as a risk factor of NEC,[4,36] and in our study of the incidence of NEC in Sweden there seemed to be a greater risk of NEC in boys than in girls[1]. In this case-control-study, however, there were 55% boys among NEC cases and controls alike. Compared to the national birth cohort, the sex distribution differed significantly in GA 32–36 weeks, where there were 57% boys in the study population and 51% nationally, p<0.001, but not in any other subgroup.

The positive association with cardiac malformation only in GA > 31 w is in keeping with the results of other studies[39–41]. In contrast, for PDA, the association was positive in all sub-groups, although statistically significant in GA > 28 w only. Unlike the findings of Lee et.al.[42], surgical treatment did not further enhance the association, but whether the PDA itself, treatment with indomethacin, or perhaps a combination of both, accounted for the enhanced risk is not possible to discern. Both have been suggested as risk factors of NEC in earlier studies[41,43].

### Neonatal complications

Red blood cell transfusion as a risk factor for NEC is under dispute. Evidence is equivocal and mostly from observational studies[44]. In the registers that we have used, the temporal relationship between red cell transfusion and NEC onset cannot be determined, so the positive association does not imply a causative relationship. It may rather mirror the general vulnerable state of infants susceptible to NEC or, as Hay et.al. suggest, even treatment given for prodromal symptoms of NEC[44] or for sepsis secondary to NEC.

Bacterial infection, including sepsis, was positively associated with NEC. Data on infection with other organisms were too infrequent to be reliably analyzed. Sepsis and bloodstream infections are well-known manifestations of NEC,[45] but have also been suggested as a contributing factor in the pathogenesis[46]. Without information on the temporal relationships of the diagnoses, the interpretation of our finding is unclear.

### Association with specific complications of prematurity

IRDS, ROP, BPD, and IVH are all morbidities specific for the premature, partially interrelated to one another, with many risk factors in common with NEC. Similar to NEC, IVH is described as a complex disorder involving changes in cerebral blood flow, coagulation, angiogenesis, and inflammation, mediated by complicated interactions of various cytokines[47]. The positive relationship between IVH, which normally occurs within the first days of life, and NEC, which rarely occurs before one week of age,[2] is, however, probably explained by pathogenetic mechanisms that predispose for both conditions but are not included in our multivariable model.

Previous results regarding associations of ROP and BPD with NEC are conflicting[27,48–50]. Our finding of a negative relationship of BPD with NEC, after correcting for confounders and common risk factors, suggests that there are other factors, not included in the model, that affect the risk of BPD and NEC differently.

### SIP contamination of the data set

It is generally accepted that spontaneous intestinal perforation (SIP) should be differentiated from NEC [7]. In this population, a discharge diagnosis of intestinal perforation in the newborn among cases may correspond to SIP that has not been separated from NEC, revised diagnoses after surgery for presumed NEC, misclassification of perforated NEC, or even the development of NEC after surgical recovery from SIP[51]. The awareness of SIP and its distinction from NEC can be assumed to have increased over time, so that the occurrence of SIP is more overt towards the end of the study period, without necessarily having increased. These considerations led to the decision not to exclude individuals with a diagnosis of intestinal perforation in the newborn.

### Strengths and limitations

The comprehensive Swedish national health care registers offer good opportunities for epidemiological research. Despite a small population and, from an international perspective, low incidence rates of NEC in Sweden, the present study represents a large number of subjects over a period of 23 years in which major advances have been made in ante- and perinatal care resulting in a significant growth of the population at greatest risk for NEC.

On evaluation, the registers used have been found to hold high quality [52]. Nevertheless, as in all register studies, the data were routinely collected for administrative reasons rather than specifically for research, which implies that the information collected cannot be influenced to fit the objective of this particular study. The inclusion of a case is based on the discharge diagnosis without any knowledge about the diagnostic criteria used in each case. Validation of the MBR has shown errors below 5% [53], which is in parity with the findings of Palleri et.al. when manually validating the discharge diagnosis of NEC in Stockholm County 2009–2014 [54]. Hence about 5% of cases can be expected not to have had NEC. The effect of such an error, however, should be dilution of the dataset, which may weaken actual associations but not introduce false ones. Nevertheless, results must be interpreted with care, baring in mind the risk of spurious associations caused by the great number of variables.

Looking for potential risk factors for NEC, timing is important, but the possibilities to discern timely relationships for events and diagnoses are extremely limited. Information on some potentially important factors such as feeding and medication is lacking all together. There is also a problem with missing values, competing risk of early death in infants that would potentially have developed NEC, and eventual difference in associations due to changes in management during the long study period. We found no clinically important difference in the sensitivity analyses related to these factors.

Missing personal identification numbers in new born infants is another problem with using the registers. The number is given to all Swedish residents at birth or immigration,[9] but during the study period, there was a delay of a few weeks before newborns received their number, resulting in a substantial proportion of discharge registrations with missing identification number, impeding linkage between individual hospitalizations belonging to one individual as well as between registers. The study population is thus reduced by 22%. In our previous study, diagnoses of prematurity, mortality and sex distribution were compared between the study population and subjects with incomplete identification without demonstrating any significant differences. The missing cases were thus estimated not to represent any systematic error or induce any substantial bias[1]. The risk for such an influence is expected to be even less with the case-control design. We obtained fewer controls in GA < 32 w due to the rarity of eligible matched controls. This potential risk for inducing bias should have been controlled for by the inclusion of GA and year of birth in all analyses.

## Conclusion

Our results support the notion that NEC is a common pathophysiological pathway of multifactorial etiology, rather than a uniform disease entity. Some of the associations identified in this study seem to be primarily related to an unspecific vulnerability, others may have more direct pathophysiologic associations with NEC, and some may affect both. Differences in management of antenatal and perinatal complications may influence the impact of certain risk factors and contribute to the disparate results of different studies.

## Supporting information

S1 Table Associations of maternal, gestational, fetal, and perinatal factors with NEC in subgroups according to gestational age, univariable regression. (DOCX)
