# Supplementary material for: Maternal, fetal and perinatal factors associated with necrotizing enterocolitis in Sweden. A national case-control study
Source: PLoS One. 2018 Mar 23;13(3):e0194352. doi: 10.1371/journal.pone.0194352 (PMC5865724; doi:10.1371/journal.pone.0194352)
Supplement: S1 Table — (DOCX) [file pone.0194352.s001.docx]

**Table 3. Associations of maternal, gestational, fetal, and perinatal factors with NEC in subgroups according to gestational age, univariable regression.**OR- Odds ratio, CI – 95% confidence interval; GA – gestation age

|  | Term | | | GA 32-36 w | | | GA 28-31 w | | | GA < 28 w | | |
| --- | --- | --- | --- | --- | --- | --- | --- | --- | --- | --- | --- | --- |
| Factor | **OR** | *CI* | pvalue | **OR** | *CI* | pvalue | **OR** | *CI* | pvalue | **OR** | *CI* | pvalue |
| Maternal age (years) | **1.03** | *1-1.07* | 0.053 | **1.05** | *1.01-1.08* | 0.014 | **1.02** | *0.99-1.05* | 0.135 | **0.98** | *0.96-1* | 0.102 |
| Maternal weight (kg) | **1** | *0.98-1.02* | 0.814 | **1** | *0.98-1.02* | 0.895 | **0.99** | *0.97-1* | 0.058 | **0.98** | *0.97-1* | 0.008 |
| Maternal smoking | **0.94** | *0.58-1.53* | 0.804 | **0.95** | *0.55-1.64* | 0.853 | **0.65** | *0.41-1.04* | 0.074 | **0.94** | *0.64-1.38* | 0.749 |
| Maternal unemployment | **1.36** | *0.93-1.98* | 0.111 | **1.53** | *1.01-2.32* | 0.047 | **1.07** | *0.77-1.48* | 0.677 | **1.06** | *0.79-1.42* | 0.711 |
| Education>12 y | **0.54** | *0.29-1* | 0.051 | **0.31** | *0.14-0.69* | 0.004 | **0.87** | *0.57-1.33* | 0.514 | **1.12** | *0.78-1.61* | 0.539 |
| Maternal disp. income (100 E) | **1.14** | *0.98-1.33* | 0.085 | **1.38** | *1.08-1.76* | 0.01 | **1.05** | *0.83-1.31* | 0.694 | **1.1** | *0.92-1.31* | 0.281 |
| Born in Stockholm | **2.2** | *1.51-3.21* | 0 | **2.31** | *1.53-3.51* | 0 | **1.83** | *1.31-2.55* | 0 | **1.79** | *1.33-2.41* | 0 |
| First born | **1.47** | *1.02-2.12* | 0.038 | **0.88** | *0.59-1.31* | 0.527 | **0.81** | *0.59-1.1* | 0.172 | **0.93** | *0.71-1.23* | 0.622 |
| Maternal diabetes | **3.85** | *1.36-10.91* | 0.011 | **0.51** | *0.12-2.17* | 0.359 | **0.83** | *0.34-1.98* | 0.669 | **0.49** | *0.17-1.4* | 0.18 |
| Preeclampsia | **0.95** | *0.46-1.98* | 0.902 | **1.23** | *0.73-2.06* | 0.431 | **0.96** | *0.68-1.34* | 0.806 | **1.2** | *0.85-1.68* | 0.295 |
| Maternal urinary infection | **1.31** | *0.71-2.39* | 0.388 | **0.83** | *0.43-1.59* | 0.574 | **0.64** | *0.37-1.1* | 0.108 | **0.57** | *0.36-0.89* | 0.015 |
| Isoimmunization | **2.86** | *1.21-6.76* | 0.017 | **1.09** | *0.44-2.69* | 0.846 | **2.24** | *1.2-4.19* | 0.011 | **0.97** | *0.49-1.94* | 0.936 |
| Placental complications | **2.89** | *1.54-5.44* | 0.001 | **1.4** | *0.83-2.38* | 0.209 | **1.19** | *0.82-1.7* | 0.358 | **1.06** | *0.75-1.49* | 0.751 |
| Chorioamnionitis | **2.04** | *0.21-19.73* | 0.54 | **1.25** | *0.27-5.78* | 0.778 | **0.39** | *0.14-1.09* | 0.071 | **0.75** | *0.49-1.16* | 0.2 |
| Premature rupture of membranes | **1.01** | *0.22-4.6* | 0.987 | **0.38** | *0.22-0.66* | 0.001 | **0.65** | *0.44-0.95* | 0.025 | **0.57** | *0.41-0.79* | 0.001 |
| Fetal distress | **6.48** | *4.05-10.36* | 0 | **3.44** | *2.2-5.38* | 0 | **1.05** | *0.73-1.5* | 0.792 | **1.43** | *1.01-2.02* | 0.047 |
| Spontaneously starting delivery | **0.54** | *0.35-0.82* | 0.004 | **0.36** | *0.23-0.57* | 0 | **0.81** | *0.58-1.13* | 0.219 | **0.85** | *0.64-1.13* | 0.256 |
| Cesarean section | **5.4** | *3.59-8.12* | 0 | **3.42** | *2.25-5.22* | 0 | **1.5** | *1.07-2.1* | 0.019 | **0.99** | *0.75-1.32* | 0.963 |
| Apgar < 7 at 1 minute | **4.09** | *2.27-7.38* | 0 | **3.01** | *1.78-5.09* | 0 | **0.94** | *0.65-1.35* | 0.741 | **1.02** | *0.75-1.37* | 0.922 |
| Apgar<7 at1 and 5 minutes | **13.08** | *3.86-44.32* | 0 | **3.39** | *1.57-7.35* | 0.002 | **1.27** | *0.76-2.14* | 0.36 | **0.85** | *0.6-1.2* | 0.359 |
| Apgar<7 at 1. 5. and 10 minutes | **67.26** | *8.51-531.91* | 0 | **0.72** | *0.15-3.33* | 0.672 | **0.44** | *0.18-1.06* | 0.068 | **0.81** | *0.51-1.3* | 0.382 |
| Male sex | **1.16** | *0.81-1.67* | 0.423 | **0.84** | *0.57-1.25* | 0.394 | **1.11** | *0.81-1.5* | 0.52 | **0.92** | *0.7-1.2* | 0.526 |
| Birth weight (100 g) | **0.93** | *0.9-1.03* | 0.001 | **0.9** | *0.86-0.95* | 0 | **0.89** | *0.84-0.94* | 0 | **0.87** | *0.8-0.94* | 0.001 |
| Length (cm) | **0.87** | *0.79-0.96* | 0.004 | **0.86** | *0.79-0.93* | 0 | **0.89** | *0.84-0.94* | 0 | **0.89** | *0.83-0.94* | 0 |
| Ponderal index | **0.91** | *0.84-0.98* | 0.009 | **0.96** | *0.89-1.03* | 0.262 | **1** | *0.93-1.07* | 0.989 | **1.03** | *0.97-1.09* | 0.359 |
| Head circumference | **0.87** | *0.76-0.99* | 0.03 | **0.92** | *0.81-1.05* | 0.226 | **0.89** | *0.81-0.97* | 0.011 | **0.92** | *0.84-1* | 0.06 |
| Small for gestational age | **7.77** | *3.78-15.97* | 0 | **2.59** | *1.58-4.25* | 0 | **1.46** | *1.05-2.01* | 0.023 | **1.24** | *0.89-1.74* | 0.202 |
| Neonatal anemia | **48.94** | *10.94-218.96* | 0 | **4.24** | *2.11-8.52* | 0 | **1.08** | *0.75-1.56* | 0.685 | **0.56** | *0.41-0.76* | 0 |
| Icterus of the newborn | **3.25** | *1.65-6.41* | 0.001 | **0.52** | *0.34-0.8* | 0.003 | **0.45** | *0.33-0.63* | 0 | **0.66** | *0.49-0.88* | 0.005 |
| Bacterial infection including sepsis | **30.75** | *13.09-72.21* | 0 | **6.45** | *3.78-11.01* | 0 | **2.76** | *1.99-3.82* | 0 | **1.34** | *1.01-1.78* | 0.041 |
| Erythrocyte transfusion | **66.81** | *8.46-527.44* | 0 | **5.18** | *2.62-10.21* | 0 | **1.78** | *1.21-2.6* | 0.003 | **0.87** | *0.64-1.2* | 0.4 |
| Persistent ductus arteriosus | **41.78** | *9.18-190.19* | 0 | **5.95** | *2.54-13.93* | 0 | **2.04** | *1.37-3.04* | 0 | **1.33** | *0.99-1.77* | 0.058 |
| Cardiac malformation | **17.31** | *7.35-40.74* | 0 | **4.19** | *2.01-8.76* | 0 | **0.88** | *0.39-1.98* | 0.751 | **0.63** | *0.31-1.31* | 0.218 |
| Gastrointestinal malformation | **44.9** | *5.48-368.06* | 0 | **2.36** | *0.62-9.03* | 0.211 | **1.49** | *0.54-4.07* | 0.439 | **3.39** | *1.2-9.62* | 0.022 |
| Chromosomal abnormality | **7.76** | *2.31-26.05* | 0.001 | **8.37** | *2.27-30.85* | 0.001 | **1.11** | *0.3-4.06* | 0.875 | **3.39** | *0.89-12.95* | 0.075 |
| Infant respiratory distress syndrome | **46.36** | *5.63-382.02* | 0 | **1.73** | *0.89-3.37* | 0.107 | **1.15** | *0.84-1.58* | 0.38 | **1.44** | *1.05-1.96* | 0.023 |
| Retinopathy of the premature |  | *-* |  | **6.38** | *0.88-46.29* | 0.067 | **1.07** | *0.4-2.86* | 0.892 | **1.18** | *0.82-1.71* | 0.374 |
| Intracranial bleeding |  | *-* |  | **6.73** | *2.1-21.61* | 0.001 | **1.33** | *0.8-2.22* | 0.269 | **1.54** | *1.13-2.1* | 0.007 |
| Bronchopulmonary dysplasia |  | *-* |  | **6.26** | *0.39-101.48* | 0.197 | **0.79** | *0.43-1.46* | 0.451 | **0.74** | *0.54-1.02* | 0.065 |
